# Influenza Vaccination Effectiveness Against Influenza-Associated Hospitalization in Children and the Effects of Repeated Vaccination

**DOI:** 10.1093/infdis/jiag047

**Published:** 2026-01-23

**Authors:** Xuan Yu, So-Lun Lee, Mike Y W Kwan, Shuyi Zhong, Caitriona Murphy, Eunice L Y Chan, Joshua S C Wong, Sheena G Sullivan, Malik Peiris, Benjamin J Cowling

**Affiliations:** World Health Organization Collaborating Centre for Infectious Disease Epidemiology and Control, School of Public Health, The University of Hong Kong, Hong Kong Special Administrative Region, China; Department of Paediatrics and Adolescent Medicine, Li Ka Shing Faculty of Medicine, The University of Hong Kong, Hong Kong Special Administrative Region, China; Department of Paediatrics and Adolescent Medicine, Princess Margaret Hospital, Hong Kong Special Administrative Region, China; World Health Organization Collaborating Centre for Infectious Disease Epidemiology and Control, School of Public Health, The University of Hong Kong, Hong Kong Special Administrative Region, China; World Health Organization Collaborating Centre for Infectious Disease Epidemiology and Control, School of Public Health, The University of Hong Kong, Hong Kong Special Administrative Region, China; Department of Paediatrics and Adolescent Medicine, Li Ka Shing Faculty of Medicine, The University of Hong Kong, Hong Kong Special Administrative Region, China; Department of Paediatrics and Adolescent Medicine, Princess Margaret Hospital, Hong Kong Special Administrative Region, China; School of Clinical Sciences, Monash University, Melbourne, VIC, Australia; Fielding School of Public Health, University of California, Los Angeles (UCLA), Los Angeles, California; Centre for Immunology & Infection, Hong Kong Science and Technology Park, New Territories, Hong Kong Special Administrative Region, China; World Health Organization Collaborating Centre for Infectious Disease Epidemiology and Control, School of Public Health, The University of Hong Kong, Hong Kong Special Administrative Region, China

**Keywords:** influenza, vaccination, vaccine effectiveness, A(H1N1)pdm09, repeat vaccination

## Abstract

**Background:**

Given concerns that influenza vaccine effectiveness (VE) might differ with repeated annual vaccination, we estimated VE against influenza-associated hospitalization and assessed repeat vaccination effects.

**Methods:**

We analyzed a test-negative design study conducted in 3 Hong Kong hospitals (October 2015 - July 2025), excluding the 2020/21 and 2021/22 seasons due to the absence of influenza circulation during the COVID-19 pandemic. Polymerase chain reaction testing was used to identify influenza virus infections. We used conditional logistic regression to estimate influenza VE overall, by influenza type/subtype, and by influenza vaccination status in the preceding year (repeat vaccination status).

**Results:**

We analyzed data on 34 237 children, among whom 5245 (15.3%) tested positive for influenza. VE against influenza-associated hospitalization was 57.2% (95% confidence interval [CI]: 52.3%, 61.6%), with subtype-specific VE estimates of 67.7% (95% CI: 61.8%, 72.7%) for A(H1N1)pdm09, 60.6% (95% CI: 50.8%, 68.5%) for influenza B, and 37.2% (95% CI: 24.7%, 47.6%) for A(H3N2). The overall ΔVE (repeated VE − current only VE) was −13.6% (95% CI: −33.2%, 3.2%), indicating lower VE among repeatedly vaccinated children.

**Conclusions:**

Current influenza vaccination programs provide substantial protection, but could be further improved by strategies that mitigate repeat vaccination effects, and further research is needed to identify such strategies.

## Teaser

In this study of influenza vaccine effectiveness against influenza-associated hospitalization in children, current-season vaccination substantially reduced the risk of influenza-associated hospitalization, while repeated vaccination was associated with attenuated vaccine effectiveness particularly for A(H3N2).

Seasonal influenza epidemics cause substantial morbidity and mortality across all age groups annually. In Hong Kong, a study spanning 1998–2013 estimated that influenza was associated with 431 respiratory deaths and 12 700 respiratory hospitalizations per year [1]. While influenza affects all ages, children are particularly vulnerable and experience a high disease burden. For both influenza A and B, younger children under 5 years of age experience higher hospitalization rates compared to older children [2–4]. This vulnerability is influenced not only by immunological factors such as lower immunity due to fewer prior infections but also by regional characteristics. Hong Kong's subtropical climate and high population density make it a hotspot for influenza virus transmission, characterized by prolonged influenza activity and unpredictable peak timing, distinct from the winter influenza peaks observed in temperate regions [5].

Influenza vaccination is an effective measure for reducing the risk of influenza virus infection and related illness [6]. The Hong Kong Department of Health provides free or subsidized influenza vaccination to priority groups including children from 6 months to 17 years of age and since 2018/19 has provided vaccination to children through a school-based program which initially covered children aged 3–11 years and then expanded in 2022/23 to include children up to 18 years of age [7]. In 2024/25, coverage was 55% in children 6 months to 5 years of age, 74% in children 6–11 years of age, and 56% in children 12–17 years of age [8]. Most doses administered are inactivated vaccines. In the context of Hong Kong's longstanding annual influenza vaccination policy for children, and given concerns that influenza vaccine effectiveness (VE) might differ among recipients of repeated influenza vaccination [9], we aimed to estimate VE against pediatric influenza-associated hospitalization over a prolonged recent period, while investigating potential repeat vaccination effects across different influenza types/subtypes.

Unlike some previous studies of influenza VE in hospitalized children in Hong Kong that adjusted for vaccination history as a confounder, we classified children according to their vaccination status in both the previous and current influenza seasons. This approach allowed us not only to estimate current-season VE but also to assess the modifying effect of repeated vaccination. In contrast to studies focusing on the impact of repeated vaccination on VE against specific influenza subtypes among adults, our study focused on this relationship specifically for influenza-associated hospitalization in children and considered variations across different influenza types/subtypes. Additionally, given the interruption in community influenza circulation during the COVID-19 pandemic, we examined whether the effect of repeated vaccination differed before and after this period.

## METHODS

### Study Design

We analyzed data from a test-negative design study conducted across 3 Hong Kong public hospitals from October 2015 to July 2025. The study enrolled children up to 17 years of age who were admitted to the pediatric ward with febrile acute respiratory illness, defined as having a fever ≥38°C and at least one respiratory symptom (eg, cough, runny nose, or sore throat) with symptom onset within 72 h prior to hospitalization [10]. The catchment populations of these hospitals include approximately 17% of children in Hong Kong, with a fairly representative distribution of socio-economics [11]. Demographic characteristics (age, sex), clinical information, and vaccination history for both current and prior influenza seasons were collected from parents or legal guardians using a standardized questionnaire. All children presenting with respiratory symptoms at admission routinely underwent laboratory testing for influenza A and B viruses and other common respiratory viruses. Routine testing for SARS-CoV-2 was added to this panel beginning in January 2020. Admission/discharge dates and laboratory results were obtained from medical records.

Current-season vaccination was defined as completion of a full vaccination series at least 2 weeks prior to hospitalization. Full vaccination was defined as follows: for individuals aged ≥9 years, 1 dose was required; for children <9 years, 2 doses (administered ≥4 weeks apart) were required if they had no prior influenza vaccination history. Otherwise, a single dose in the current season was considered sufficient. Children who received prior-season vaccination were defined as those with receipt of ≥1 dose of seasonal influenza vaccine during the season immediately preceding the current study period. In our study, children under age 9 who, based on this criterion, required 2 doses but received only 1 were classified as partially vaccinated. A small number of children meeting this definition of partial vaccination were consequently excluded from the subsequent analyses [4, 12]. Considering the potential association between influenza vaccination and SARS-CoV-2 vaccination, children testing negative for influenza but positive for SARS-CoV-2 were excluded from control groups [13]. Additionally, as the analysis required complete vaccination history, children below 1 year were excluded due to the limited opportunity for prior influenza vaccination. Further exclusions involved partially vaccinated participants and influenza C–positive cases.

### Ethical Approval

The study was approved by the Institutional Review Board of the University of Hong Kong (ref: UW 09-279) and the Kowloon West Cluster Research Ethics Committee (ref: KW/FR-15-134(89-14)). Verbal informed consent was obtained from participants' parents or legal guardians.

### Statistical Analysis

Baseline characteristics were compared between children with different influenza vaccination statuses using χ² tests. As the Hong Kong Department of Health's vaccination policy, including school-based programs, typically commences in October or November each year, both the availability of influenza vaccines with the latest formulations and the majority of influenza vaccinations occur during this period. Therefore, we defined epidemic periods as October in each year to September of the following year to evaluate the protective effects of vaccination against influenza-associated hospitalization. When classifying individual epidemics in a particular year, to avoid small sample size issues in individual seasons, we specified a requirement of ≥50 cases per seasonal period for a particular type/subtype to be included in type/subtype-specific analyses.

We used conditional logistic regression models, matched on 2-week calendar periods, to estimate adjusted odds ratios for influenza positivity by vaccination status, adjusting for age (using a spline function), sex, and admitting hospital. Vaccination status was categorized into 4 groups based on vaccination history in the previous and current influenza seasons: *never vaccinated* (unvaccinated in both seasons), *prior-season only* (vaccinated in the previous season only), *current-season only* (vaccinated in the current season only), and *repeat vaccination* (vaccinated in both seasons). Based on this categorical variable, VE was estimated for each of the 3 vaccinated groups as (1 − adjusted odds ratio) × 100%, using the never-vaccinated group as the reference. Overall and type/subtype-specific VE were also estimated through stratified analyses by influenza type/subtype. Corresponding 95% confidence intervals (CIs) were computed using the standard errors estimated from this model. Given that repeat vaccination effects may vary by season and subtype and that these effects can be influenced by the antigenic match between seasonal vaccine strains and circulating viruses, we conducted stratified analyses by individual influenza season within each subtype. All statistical analyses were conducted in R version 4.3.1 (R Foundation for Statistical Computing, Vienna, Austria).

## RESULTS

**Figure 1.**
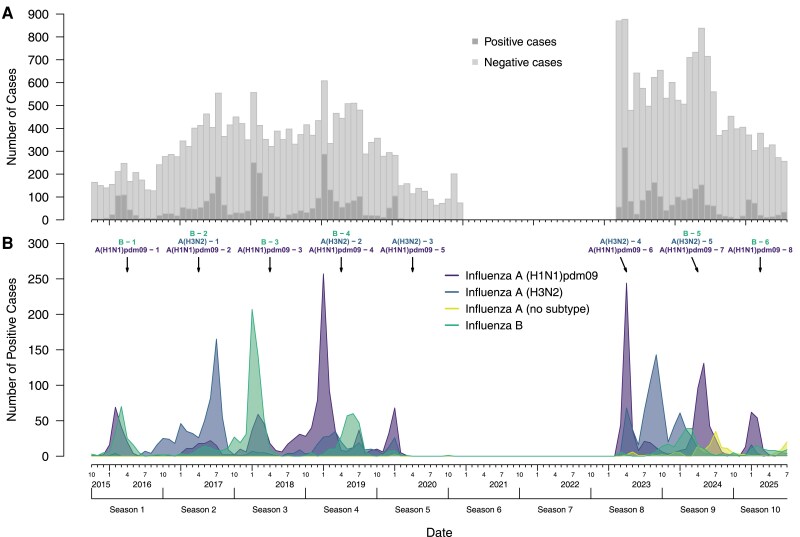
Number of hospitalized children enrolled over time from October 2015 to March 2025, with stacked bars indicating influenza test results. Influenza did not circulate in the community between March 2020 and February 2023, and this period is excluded from our study.

We analyzed data from patients admitted from October 2015 through to July 2025. Multiple influenza types/subtypes circulated in most years. However, influenza circulation was absent in the community during 3 years of the COVID-19 pandemic [14], leading to the suspension of influenza surveillance for children hospitalized in the 3 hospitals under study from January 2021 to February 2023. Consequently, the 2019/20 epidemic period comprised only the winter influenza wave (October 2019 through February 2020), and the 2022/23 season only included circulation between March and September 2023 (Figure 1).

From 1 October 2015 to 31 July 2025, 34 919 children hospitalized with febrile acute respiratory symptoms were enrolled. Among influenza-negative participants, 682 tested positive for SARS-CoV-2 and were excluded. Of the remaining 34 237 children aged 1–17 years (Table 1), 5245 (15.3%) tested positive for influenza, comprising 2149 (41.0%) influenza A(H1N1)pdm09 cases, 1675 (31.9%) influenza A(H3N2) cases, and 1265 (24.1%) influenza B cases, with <3% unknown subtypes. Influenza vaccination was reported by 12 071 children (35.3% of total), with 11 350 (94.0%) receiving quadrivalent formulations, 394 (3.3%) receiving trivalent formulations, and the remaining 327 (2.7%) receiving unknown-type vaccines. Among the vaccinated children, 8667 (71.8%) were also vaccinated during the previous influenza season. Among the 22 166 unvaccinated children (64.7% of total), only 1394 (6.3%) had received prior-season vaccination. Before the implementation of the school-based influenza vaccination program in the 2018/19 season, vaccine coverage among hospitalized children was 20.8%. It increased to 42.4% thereafter. From the 2019/20 season onward, the proportion of children receiving repeated vaccination (vaccinated in both the previous and current influenza seasons) rose from 16.8% to 41.2% (Supplementary Table 1), indicating that the vaccination program had substantially increased both overall coverage and the uptake of repeated vaccination among children. The analysis of overall VE included these 34 237 children. For type/subtype-specific analyses, we identified 19 distinct influenza epidemics including 8 A(H1N1)pdm09, 5 A(H3N2), and 6 B epidemics, during which there were subsets of 2136 A(H1N1)pdm09, 1562 A(H3N2), and 1208 influenza B cases, respectively.

**Table 1.** Characteristics of Children Admitted to Hospital With Febrile Acute Respiratory Illness and Included in This Study

Characteristic	Overall (n = 34 237)	Repeat Vaccination Status											
		Repeat Vaccination (n = 8667)			Current-Season Only (n = 3404)			Prior-Season Only (n = 1394)			Never Vaccinated (n = 20 772)		
		Cases	Controls	*P* Value	Cases	Controls	*P* Value	Cases	Controls	*P* Value	Cases	Controls	*P* Value
Sex													
Male	18 733	481	4199	0.704	132	1750	.772	76	691	.017	2114	9290	.026
Female	15 504	399	3588		102	1420		89	538		1852	7516	
Age group													
1–3 y	16 423	83	1538	<.001	132	2173	<.001	45	354	.467	1829	10 269	<.001
4–8 y	12 269	509	4394		88	821		79	621		1487	4270	
9–17 y	5545	288	1855		14	176		41	254		650	2267	
Influenza season													
2015/16	2115	30	328	<.001	11	184	<.001	14	60	<.001	299	1189	<.001
2016/17	4520	46	417		39	288		13	144		651	2922	
2017/18	4704	50	468		20	480		23	103		703	2857	
2018/19	5197	116	776		65	625		27	161		822	2605	
2019/20	2120	28	422		11	235		13	117		141	1153	
2022/23	5146	231	1270		22	432		29	118		603	2441	
2023/24	6955	282	2658		57	639		35	304		569	2411	
2024/25	3480	97	1448		9	287		11	222		178	1228	

**Figure 2.**
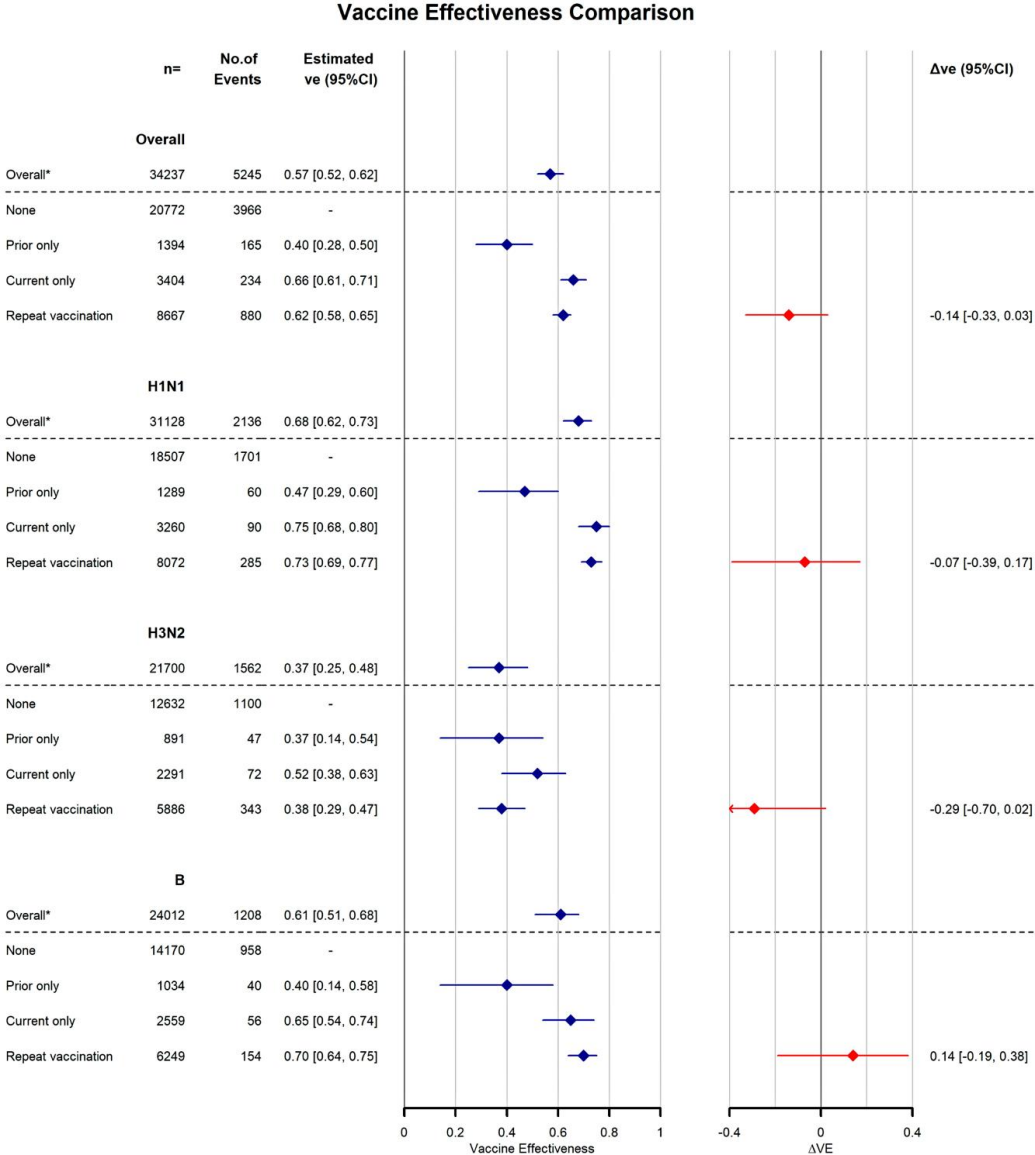
Estimated vaccine effectiveness against influenza-associated hospitalization against all influenza and against influenza A(H1N1)pdm09, A(H3N2), and B, stratified by repeated vaccination status. Differences in VE and corresponding 95% CIs were estimated via bootstrapping.

Vaccine effectiveness against influenza-associated hospitalization was estimated as 57.2% (95% CI: 52.3%, 61.6%) overall. The estimated subtype-specific VE for A(H1N1)pdm09 was 67.7% (95% CI: 61.8%, 72.7%), slightly higher than that for B (60.6%, 95% CI: 50.8%, 68.5%), while the estimated VE for A(H3N2) was lower at 37.2% (95% CI: 24.7%, 47.6%). When categorizing by prior- and current-season vaccination status, the overall VE was 66.1% (95% CI: 60.9%, 70.7%) for children vaccinated in the current season only, compared to 61.5% (95% CI: 57.9%, 64.8%) for repeat-vaccinated children, indicating a slightly lower effectiveness in the latter group. Children vaccinated only in the prior season showed substantially lower VE of 40.2% (95% CI: 28.4%, 50.1%) (Figure 2). A similar pattern was observed for influenza A(H1N1)pdm09. For influenza A(H3N2), VE in repeat-vaccinated children (38.2%; 95% CI: 28.6%, 46.5%) was lower than that in children vaccinated in the current season only and similar to that in prior-season-only vaccinees (37.2%; 95% CI: 13.9%, 54.2%), yielding an effectiveness similar to that provided by prior-season vaccination alone. In contrast, for influenza B, repeat-vaccinated children showed higher VE (70.2%; 95% CI: 63.9%, 75.4%) compared to all single-season vaccination groups.

Analysis using dummy variables in a single conditional logistic regression model estimated the overall ΔVE (VE_repeated − VE_current only) of −13.6% (95% CI: −33.2%, 3.2%). This pattern of lower VE estimates with repeated vaccination persisted across all influenza A subtypes (Figure 2), with a particularly pronounced ΔVE of −29.1% (95% CI: −69.9%, 1.8%) observed for A(H3N2), although this estimate did not reach statistical significance. This suggests that while repeat vaccination was associated with a modest reduction in VE against A(H1N1)pdm09 (−7.4%; 95% CI: −38.8%, 16.9%), it was associated with a more substantial loss of protection against influenza A(H3N2). In contrast, for influenza B, repeated vaccination was associated with a positive ΔVE of 14.2% (95% CI: −19.0%, 38.2%).

**Figure 3.**
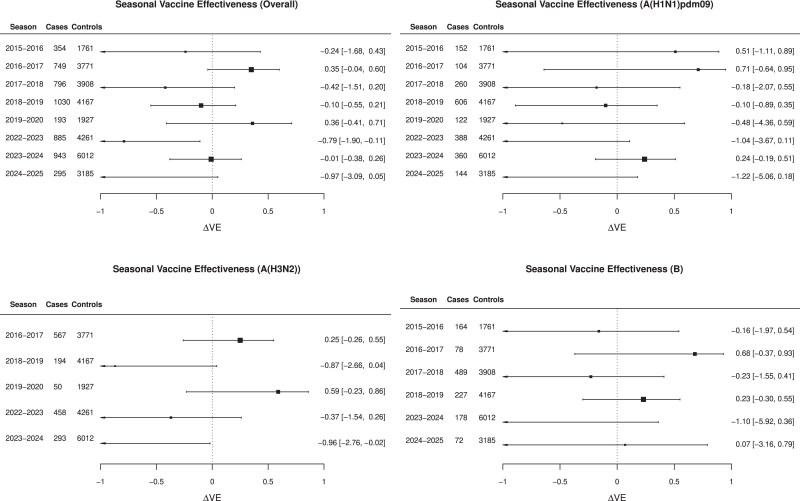
Season-specific ΔVE estimates for vaccination against overall influenza and against influenza A(H1N1)pdm09, A(H3N2), and B.

Stratified analyses by individual influenza season and subtype (Figure 3) revealed notable variations in the effect of repeat vaccination. The 2018/19 season represented the most severe pediatric epidemic in Hong Kong in at least the past decade, with an overall ΔVE of −10.3% (95% CI: −54.8%–21.4%). Influenza A(H1N1)pdm09 was the predominant circulating subtype, and repeat vaccination was similarly associated with reduced effectiveness (ΔVE = −10.4%; 95% CI: −88.8%–35.4%). Subsequent seasons with considerable influenza activity, 2022/23 and 2023/24, also yielded negative point estimates for overall ΔVE. However, unlike the nonsignificant estimate in 2023/24 (−1.2%; 95% CI: −38.2%–25.9%), in which timely vaccine strain updates were associated with favorable performance of repeated vaccination against A(H1N1)pdm09, there was a more pronounced effect in the 2022/23 season (ΔVE = −79.5%; 95% CI: −190.0% to −11.1%). This season was characterized by co-circulation of A(H1N1)pdm09 and A(H3N2), with corresponding ΔVE estimates of −104.2% (95% CI: −366.7%–10.7%) and −37.0% (95% CI: −153.9%–26.1%), respectively. The estimate for A(H1N1)pdm09 suggests a substantial reduction in VE associated with repeat vaccination. For influenza B, the most severe season was 2017/18, during which the estimated ΔVE was −22.8% (95% CI: −155.2%–40.9%). Notably, A(H1N1)pdm09, which also circulated that season, was associated with a negative ΔVE (−18.0%; 95% CI: −207.3%–54.7%). This pattern may indicate that the effect of repeated vaccination reflects a trade-off between the benefits of antigenic matching and the interference arising from the co-circulation of both influenza types/subtypes.

## DISCUSSION

Our findings indicate that current-season vaccination substantially reduces the risk of influenza-associated hospitalization in children (VE = 57.2%; 95% CI: 52.3%, 61.6%). Vaccine effectiveness estimates for children vaccinated in the current season, whether as a single dose or repeated vaccination, were consistently comparable to or higher than those for children vaccinated only in the previous season. Furthermore, regardless of the specific strategy, vaccination conferred a protective benefit compared with not being vaccinated. However, the effect of repeated vaccination appeared to differ by virus subtype and season, a pattern particularly evident for influenza A(H3N2).

Previous systematic reviews and meta-analyses have reported that current-season vaccination generally confers substantial protection against medically attended influenza or hospitalization regardless of prior vaccination history. Conversely, protection is typically lowest among those vaccinated only in prior seasons. This observation is consistent with the low VE observed in the group vaccinated only in the previous season in our study [15, 16]. Comparisons between children vaccinated in both current and prior seasons versus those vaccinated only in the current season have yielded mixed results. Our findings are consistent with several other observations of repeat vaccination effects. These studies similarly indicate a non-statistically significant reduction in VE for A(H1N1)pdm09 [15] and a more pronounced reduction for A(H3N2) among repeat vaccinees [17, 18] while showing higher protection against influenza B [19]. However, other observations have differed. For example, results from Canada during the 2011/12 season showed higher VE for both influenza A and B types among repeat vaccinees compared to those vaccinated in a single season only [20]. Additionally, observational studies conducted in Marshfield, Wisconsin, from 2004/05 through 2012/13 indicated that repeatedly vaccinated individuals had lower influenza B VE compared to those vaccinated in single seasons [21].

Consistent with the prior literature, we observed markedly lower VE against influenza A(H3N2) compared with A(H1N1)pdm09 and influenza B [4, 22–24], alongside a more pronounced negative ΔVE for A(H3N2). The comparatively lower protection associated with repeated vaccination for A(H3N2) could be linked to the rapid antigenic drift of the hemagglutinin protein [25], which occurs more quickly in this subtype than in others [26]. This rapid evolution increases the likelihood of mismatch between vaccine strains and circulating variants, facilitating the evasion of vaccine-induced immunity [27, 28]. These viral characteristics support the “antigenic distance hypothesis,” which suggests that negative interference from repeated vaccination is strongest when the current vaccine strain remains antigenically similar to prior strains but distinct from the circulating virus [29]. Furthermore, repeated exposure to related but mismatched H3N2 antigens may amplify “immune imprinting” (or original antigenic sin) [30, 31]. By reinforcing antibody responses to older epitopes at the expense of coverage against novel sites, this mechanism offers a plausible explanation for the pronounced repeat vaccination effects observed for A(H3N2).

In contrast to influenza A, we observed higher VE against influenza B among repeatedly vaccinated children compared with all single-season vaccination groups, resulting in a positive ΔVE. This finding aligns with several analyses reporting a modest benefit for repeat vaccination in influenza B [19, 32]. However, it is important to note that these effects were not uniform across all periods; season-specific estimates revealed variability, with half of the 6 studied seasons demonstrating enhanced effectiveness from repeated vaccination (Figure 3). Notably, during the 2018/19 epidemic, repeated vaccination conferred a marked increase in protection against influenza B. Several factors may explain this benefit. Influenza B generally exhibits slower antigenic drift and a more stable lineage structure, which may facilitate the accumulation of cross-protective immunity through repeated vaccination rather than driving detrimental imprinting [33, 34]. Furthermore, the transition from trivalent to quadrivalent vaccines, which provide coverage against both B/Victoria and B/Yamagata lineages [35], may enhance cross-lineage protection over multiple seasons, supporting the higher VE observed in repeatedly vaccinated children in this study.

The stratified analyses show substantial variability in ΔVE across seasons. Overall, ΔVE estimates were negative for the majority of influenza seasons, with more pronounced negative point estimates observed during seasons characterized by the co-circulation of multiple influenza A subtypes or both influenza A and B. This pattern is consistent with reports suggesting that the modifying effects of repeated vaccination are amplified in antigenically mismatched or complex multi-subtype seasons [36]. Co-circulation increases the complexity of individual exposure histories by combining repeated vaccination with natural infections and potentially unmeasured confounding [37, 38]. This complexity may influence VE estimates and widen CIs, as evidenced by the highly imprecise ΔVE values observed for A(H1N1)pdm09 and A(H3N2) during the 2022/23 season and for A(H1N1)pdm09 and B during the 2024/25 season (Figure 3).

Data collection was interrupted from early 2020 to early 2023 due to COVID-19 containment policies [14]. The resurgence of influenza in 2023 was accompanied by type- and subtype-specific VE estimates that were somewhat higher than the longer-term trend [7]. When comparing VE before and after the onset of the COVID-19 pandemic (2019/20 season; Supplementary Table 2), VE estimates among children vaccinated only in the current season were generally higher after the pandemic than before, except for A(H1N1)pdm09, for which VE had already been relatively high in the pre-pandemic period.

In additional exploratory analyses stratified by the implementation of the school-based influenza vaccination program (2018/19 season; Supplementary Table 3), VE among children vaccinated only in the current season showed little change after the program was introduced, except for A(H3N2). In contrast, VE among those vaccinated only in the prior season changed markedly for influenza A(H3N2) and B, albeit in opposite directions. The overall ΔVE shifted from 6.6% (95% CI: −29.2%–32.5%) before implementation to −19.1% (95% CI: −43.5%–1.2%) afterward, again suggesting a tendency toward more negative estimates. Higher influenza vaccine coverage among school-aged children may confer indirect protection to unvaccinated children and thereby attenuate observed differences between vaccination history groups.

This study has several limitations. First, as a hospital-based TND, our VE estimates may not be directly generalizable to all children in the community, because hospitalized children (both cases and controls) are likely to have a higher burden of underlying conditions or more severe illness than those who are not hospitalized. These differences in baseline health status and in clinical admission and hospital utilization patterns across subgroups may affect both VE and vaccination histories, even though the test-negative design helps to mitigate confounding related to healthcare-seeking among hospitalized patients with similar acute respiratory symptoms. These factors may therefore limit extrapolation of our findings to the broader pediatric population [39–41]. Second, while we adjusted for potential confounders of vaccination and infection in our analysis, the full causal structure for repeat vaccination may be more complex than accounted for in our analysis [42]. Nevertheless, some studies have suggested this confounding may not significantly affect the results [32]. Third, the period-stratified comparisons by pre– versus post–COVID-19 pandemic and by pre– versus post–school-based vaccination program were based on a limited number of seasons and should be interpreted as exploratory, post hoc re-groupings of the underlying season-specific estimates rather than primary analyses. The 2 temporal groupings were derived from the same long-term dataset and therefore involve partially overlapping periods, which further complicates interpretation of ΔVE differences between groupings. Changes in virus circulation, population immunity, and healthcare-seeking behavior across these periods may have introduced residual confounding that could not be fully controlled. Finally, small sample sizes in some season-subtype strata resulted in wide CIs and reduced statistical precision, and the multiple subgroup and period comparisons raise the possibility that some apparent differences reflect sparse data or statistical fluctuation rather than true heterogeneity in repeated-vaccination effects.

In conclusion, our analysis of hospitalized children in Hong Kong, encompassing 19 distinct influenza epidemics, revealed a VE of 57.2% (95% CI: 52.3%, 61.6%) against influenza-associated hospitalization across all subtypes and periods. Vaccine effectiveness estimates differed substantially by subtype: 67.7% for A(H1N1)pdm09, 60.6% for B, and 37.2% for A(H3N2). Repeated vaccination was associated with an average reduction of −13.6% in VE compared to current-season-only vaccination, with greater reductions for A(H3N2) among repeat vaccinees. Nevertheless, current vaccination programs provide substantial protection, as all vaccination strategies reduce the risk of influenza-associated hospitalization relative to no vaccination, indicating that existing policies remain effective and feasible for children. Current vaccination policies remain effective but could be further optimized to maximize protection while limiting potential attenuation from repeated vaccination. Future research could explore the introduction of enhanced vaccines [43], or alternative vaccination strategies such as tailoring booster intervals or alternating vaccine types across seasons. Finally, improving the timeliness and antigenic fit of strain selection remains critical, given the strong influence of antigenic distance on repeated-vaccination effects, particularly for A(H3N2).

## Supplementary Material

jiag047_Supplementary_Data

## Supplementary Data

Supplementary materials are available at *The Journal of Infectious Diseases* online (http://jid.oxfordjournals.org/). Supplementary materials consist of data provided by the author that are published to benefit the reader. The posted materials are not copyedited. The contents of all supplementary data are the sole responsibility of the authors. Questions or messages regarding errors should be addressed to the author.
