# Supplementary material for: Influenza Vaccination Effectiveness Against Influenza-Associated Hospitalization in Children and the Effects of Repeated Vaccination
Source: J Infect Dis. 2026 Jan 23;233(6):e1355–63. doi: 10.1093/infdis/jiag047 (PMC13271378; doi:10.1093/infdis/jiag047)
Supplement: jiag047_Supplementary_Data [file jiag047_supplementary_data.pdf]

Appendix

Table 1. Vaccination status among children admitted to hospital with febrile acute respiratory illness and included in this study

Characteristic	Vaccination Status		Repeat Vaccination Status	
	Current-Season Vaccinated	Current-Season Unvaccinated	Repeat Vaccination	Not Repeat Vaccination
**Sex**				
Male	6562 (35.0%)	12,171 (65.0%)	4680 (25.0%)	14,053 (75.0%)
Female	5509 (35.5%)	9995 (64.5%)	3987 (25.7%)	11,517 (74.3%)
**Age group**				
1 to 3 years	3926 (23.9%)	12,496 (76.1%)	1621 (9.9%)	14,802 (90.1%)
4 to 8 years	5812 (47.4%)	6457 (52.6%)	4903 (40.0%)	7366 (60.0%)
9 to 17 years	2333 (42.1%)	3212 (57.9%)	2143 (38.6%)	3402 (61.4%)
**Influenza season**				
2015/16	553 (26.1%)	1562 (73.9%)	358 (16.9%)	1757 (83.1%)
2016/17	790 (17.5%)	3730 (82.5%)	463 (10.2%)	4057 (89.8%)
2017/18	1018 (21.6%)	3686 (78.4%)	518 (11.0%)	4186 (89.0%)
2018/19	1582 (30.4%)	3615 (69.6%)	892 (17.2%)	4305 (82.8%)
2019/20	696 (32.8%)	1424 (67.2%)	450 (21.2%)	1670 (78.8%)
2022/23	1955 (38.0%)	3191 (62.0%)	1501 (29.2%)	3645 (70.8%)
2023/24	3636 (52.3%)	3319 (47.7%)	2940 (42.3%)	4015 (57.7%)
2024/25	1841 (52.9%)	1639 (47.1%)	1545 (44.4%)	1935 (55.6%)

Table 2. VE before and after the onset of the COVID-19 pandemic (2019/20 season) for overall influenza and for A(H1N1)pdm09, A(H3N2), and B separately.

Group	N	Events	VE	VE_lower	VE_upper	p_value	ΔVE	ΔVE_lower	ΔVE_upper	ΔVE_p_value
**Overall**										
Prior-covid	18656	3122								
Never Vaccinated	13342	2616	0	NA	NA	NA				
Previous-Season Only	675	90	42.3565	26.2861	54.9234	1.13E-05				
Current-Season Only	1958	146	65.311	58.2501	71.1778	4.06E-29				
repeated	2681	270	66.2361	60.9272	70.8237	4.19E-48	**2.6669**	-21.7639	22.1959	0.812982112
After-covid	15581	2123								
Never Vaccinated	7430	1350	0	NA	NA	NA				
Previous-Season Only	719	75	39.7761	21.4164	53.8464	0.000188				
Current-Season Only	1446	88	66.4334	57.7038	73.3613	2.13E-20				
repeated	5986	610	57.9241	52.6441	62.6155	1.02E-46	**-25.3504**	-60.0553	1.8294	0.069996631
**A(H1N1)pdm09**										
Prior-covid	16778	1244								
Never Vaccinated	11813	1087	0	NA	NA	NA				
Previous-Season Only	618	33	53.7679	31.7608	68.6776	0.000103				
Current-Season Only	1856	44	78.9505	70.9613	84.7417	2.26E-21				
repeated	2491	80	79.2985	73.3596	83.9135	1.93E-34	**1.6534**	-45.7967	33.6607	0.933852613
After-covid	14350	892								
Never Vaccinated	6694	614	0	NA	NA	NA				
Previous-Season Only	671	27	38.9025	5.9853	60.2945	0.025047				
Current-Season Only	1404	46	68.1889	56.205	76.8936	2.19E-12				
repeated	5581	205	67.1609	60.2006	72.9039	7.10E-30	**-3.2318**	-46.8772	27.4441	0.859669
**A(H3N2)**										
Prior-covid	10676	811								
Never Vaccinated	7340	660	0	NA	NA	NA				
Previous-Season Only	438	16	54.504	23.3982	72.9787	0.003049				
Current-Season Only	1196	48	45.1315	25.0332	59.8415	0.000164				
repeated	1702	87	45.189	29.8807	57.1551	1.71E-06	**0.1047**	-45.4768	31.4044	0.995641
After-covid	11024	751								
Never Vaccinated	5292	440	0	NA	NA	NA				
Previous-Season Only	453	31	27.2608	-9.1843	51.5407	0.124544				
Current-Season Only	1095	24	61.5738	40.9501	74.9945	1.28E-05				
repeated	4184	256	38.5259	25.7789	49.0836	4.17E-07	**-59.9796**	-150.191	-2.2956	0.039448
**B**										
Prior-covid	14565	958								
Never Vaccinated	10353	780	0	NA	NA	NA				
Previous-Season Only	501	33	17.6358	-23.842	45.2216	0.351139				
Current-Season Only	1628	51	56.865	41.1341	68.3921	1.16E-07				
repeated	2083	94	62.3332	51.9645	70.4638	3.54E-15	**12.677**	-26.5712	39.7548	0.474129
After-covid	9447	250								
Never Vaccinated	3817	178	0	NA	NA	NA				
Previous-Season Only	533	7	75.8685	47.2377	88.9631	0.000368				
Current-Season Only	931	5	87.1966	68.4535	94.8037	7.91E-06				
repeated	4166	60	79.1417	71.3731	84.802	2.91E-22	**-62.913**	-313.109	35.754	0.30394

Table 3. VE before and after the implementation of the school-based influenza vaccination program (2018/19 season) for overall influenza and for A(H1N1)pdm09, A(H3N2), and B separately.

Group	N	Events	VE	VE_lower	VE_upper	p_value	ΔVE	ΔVE_lower	ΔVE_upper	ΔVE_p_value
**Overall**										
Prior-program	11339	1899								
Never Vaccinated	8621	1653	0	NA	NA	NA				
Previous-Season Only	357	50	33.2902	7.1458	52.0733	0.01642				
Current-Season Only	1022	70	64.6709	53.9564	72.8921	1.37E-14				
repeated	1339	126	67.0118	59.3668	73.2183	1.83E-25	**6.6259**	-29.2167	32.5264	0.679164738
After-program	22898	3346								
Never Vaccinated	12151	2313	0	NA	NA	NA				
Previous-Season Only	1037	115	42.8782	29.2022	53.9125	3.16E-07				
Current-Season Only	2382	164	66.3936	60.07	71.7157	2.73E-35				
repeated	7328	754	59.9747	55.7324	63.8105	5.43E-71	**-19.1002**	-43.5476	1.1836	0.066505419
**A(H1N1)pdm09**										
Prior-program	9956	516								
Never Vaccinated	7440	472	0	NA	NA	NA				
Previous-Season Only	318	11	39.5905	-15.1191	68.2997	0.125514				
Current-Season Only	967	15	79.3079	64.7008	87.8705	7.41E-09				
repeated	1231	18	85.7084	76.5839	91.2773	1.14E-14	**30.9319**	-40.5654	66.0627	0.307355
After-program	21172	1620								
Never Vaccinated	11067	1229	0	NA	NA	NA				
Previous-Season Only	971	49	47.7544	27.9826	62.098	7.35E-05				
Current-Season Only	2293	75	73.5922	66.0284	79.472	3.70E-25				
repeated	6841	267	70.2464	65.1138	74.6239	2.11E-50	**-12.6699**	-49.3345	14.9928	0.406579
**A(H3N2)**										
Prior-program	4338	567								
Never Vaccinated	3413	491	0	NA	NA	NA				
Previous-Season Only	149	5	77.2887	43.653	90.846	0.001387				
Current-Season Only	318	30	35.0891	2.8816	56.6155	0.035532				
repeated	458	41	51.1857	30.7201	65.6057	5.96E-05	**24.7981**	-25.9246	55.0896	0.278556
After-program	17362	995								
Never Vaccinated	9219	609	0	NA	NA	NA				
Previous-Season Only	742	42	23.142	-8.3325	45.4721	0.132843				
Current-Season Only	1973	42	59.1675	43.4575	70.5126	6.92E-08				
repeated	5428	302	37.1267	25.8849	46.6633	3.22E-08	**-53.9787**	-117.082	-9.2187	0.013769
**B**										
Prior-program	10171	731								
Never Vaccinated	7589	621	0	NA	NA	NA				
Previous-Season Only	335	28	4.8291	-50.8842	39.9705	0.833269				
Current-Season Only	976	24	66.104	46.9178	78.3555	2.27E-06				
repeated	1271	58	65.3138	52.6197	74.6069	2.84E-11	**-2.3313**	-72.7415	39.3794	0.931251
After-program	13841	477								
Never Vaccinated	6581	337	0	NA	NA	NA				
Previous-Season Only	699	12	66.5451	39.0573	81.6347	0.000346				
Current-Season Only	1583	32	64.2128	47.7577	75.4849	1.02E-07				
repeated	4978	96	73.0189	65.4056	78.9568	5.14E-25	**24.607**	-14.9412	50.5477	0.189253
